# *Bombyx mori* Nuclear Polyhedrosis Virus (BmNPV) Induces Host Cell Autophagy to Benefit Infection

**DOI:** 10.3390/v10010014

**Published:** 2017-12-30

**Authors:** La Wang, Qin Xiao, Xiao-Lin Zhou, Yan Zhu, Zhan-Qi Dong, Peng Chen, Min-Hui Pan, Cheng Lu

**Affiliations:** 1 State Key Laboratory of Silkworm Genome Biology, Southwest University, Chongqing 400716, China; wangla666@163.com (L.W.); XZ953179866@163.com (Q.X.); zhouxl126@126.com (X.-L.Z.); yanxiyanxi0806@126.com (Y.Z.); zqdong@swu.edu.cn (Z.-Q.D.); PJchen@swu.edu.cn (P.C.); 2 Key Laboratory for Sericulture Functional Genomics and Biotechnology of Agricultural Ministry, Southwest University, Chongqing 400716, China

**Keywords:** *Bombyx mori*, autophagy, nuclear polyhedrosis virus (NPV), infection, autophagy related genes (*Atgs*)

## Abstract

*Bombyx mori* nuclear polyhedrosis virus (BmNPV) is an important pathogen of silkworms. Despite extensive studies in recent decades, the interaction between BmNPV and host cells is still not clearly understood. Autophagy is an intrinsic innate immune mechanism and it controls infection autonomously in virus-infected cells. In this study, we found that BmNPV infection could trigger autophagy, as demonstrated by the formation of autophagosomes, fluorescent Autophagy-related gene 8-Green Fluorescent Protein (ATG8-GFP) punctate, and lipidated ATG8. Meanwhile, autophagic flux increased significantly when monitored by the ATG8-GFP-Red Fluorescent Protein (RFP) autophagy tandem sensor and protein degradation of p62. In addition, almost all of the identified autophagy-related genes (*Atgs*) had been up-regulated post infection in mRNA levels. Then, we screened *Atgs* with the greatest fold-change during virus infection. Interestingly, all of the screened *Atgs* positively regulated the expression of virus genes. Further studies showed that *Atg7* and *Atg9* could contribute to the level of autophagy caused by viral infection. Our results demonstrated that BmNPV induced host cell autophagy to benefit its infection. These results offer insight into the complex interactions between virus and host cell, and viral pathogenesis.

## 1. Introduction

Baculoviruses are a family of DNA viruses that have a large, circular, supercoiled, and double-stranded DNA-containing genome that infect insects, particularly of the order Lepidoptera [1,2]. *Bombyx mori* nucleopolyhedrovirus (BmNPV), one of the best characterized baculoviruses, has two virion phenotypes during its infectious life cycle: occlusion-derived virus (ODV) and budded virus (BV). ODV is responsible for lateral transmission between individuals, while BV is responsible for the spread of the infection throughout the host [3,4,5]. Viruses exploit multiple mechanisms to modulate host cells to promote their replication and proliferation. Among them, autophagy mediates a highly conserved and regulated self-degradation process that is initiated as an adaptive response in unfavorable conditions, such as nutrient deprivation and innate immunity [6,7].

Autophagy is a highly conserved cell degradation pathway, but the amount of degradation varies by cell type, nutrients, environment, or cellular stresses, including virus infection [8]. The autophagy process contains three different steps: autophagy induction; cargo recognition and packaging; and autophagosome formation and degradation [9]. Several protein complexes participate and control these steps. The unc-51 like autophagy activating kinase 1 (ULK1)/Autophagy-related 1 (ATG1)—Autophagy-related 13 (ATG13) protein kinase complex controls the initiation of autophagosomes; the ATG6-vacuolar protein sorting 34 (Vps34) complex mediates the autophagosome nucleation; two ubiquitin-like conjugation systems, which are ATG12–ATG5-ATG16L1 and ATG8–II (the cleaved and lipidated form of ATG8) conjugates, regulate autophagosome expansion and completion [9]. Most Atgs are first discovered in yeast and well-conserved from yeast to insects to humans [10,11].

There has been a great deal of research into the relationship between viral infection and host autophagy. Autophagy commonly serves as a defense mechanism against viral infection [12,13]; however, many viruses have evolved to escape, or even to exploit, this mechanism to favor their survival or replication in different ways [6,14,15,16,17]. Thus, the role of autophagy in host-virus interactions is diverse for different viruses. There are few related reports about BmNPV. Inhibition of autophagy by 3-methyladenine (3-MA) dramatically results in a decrease of polyhedrin expression and polyhedra particle production, implying the involvement of polyhedrin in cellular autophagy [18]. Thus, we speculated that autophagy is likely to be involved in BmNPV infection. However, the role of autophagy during BmNPV infection and replication is still unknown. In addition, there are few studies examining the relationship between viruses and autophagy directly from the *Atgs*, so we attempted, in this aspect, to explore the role of autophagy in virus infection.

Here, we carried out our research with the phenomenon of autophagy induced by early viral infection. Then, through a series of research methods, we proved that the host autophagy induced by the virus actually paves the way for viral proliferation and replication. Furthermore, we found that autophagy-related genes could directly affect viral infection. A better understanding of the interaction between BmNPV and autophagic responses of hosts will provide new insights into viral pathogenesis.

## 2. Materials and Methods

### 2.1. Cells, Viruses, and Plasmids

The BmN-SWU1 cell line, which was derived from *Bombyx mori* ovarian tissue [19], was maintained at 27 °C in TC-100 insect medium (United States Biological, Swampscott, MA, USA), supplemented with 10% (*v*/*v*) fetal bovine serum (FBS) (Biological Industries, Beit Haemek, State of Israel), penicillin G (200 U/mL), and streptomycin sulfate (200 U/mL). *Bombyx mori* nucleopolyhedrovirus (BmNPV) (GenBank No. NC001962.1) was reproduced in BmN-SWU1 cells. The plasmids and primers used in the experiments are listed in Table 1.

### 2.2. Virus Infection and Plasmid Transfection

Monolayer cultures of BmN-SWU1 cells (10^6^ cells/well) were seeded in six- and 24-well plates (3516 and 3524, Costar Corning, New York, NY, USA) for 6 h. BmNPV was added in the cells at a multiplicity of infection (MOI) of 1 (100 μL /well in six-well plates) for autophagy induction and the supernatant was removed after 2 h of absorption. Then, fresh complete medium was added at 27 °C after rinsing with sterile phosphate-buffered saline (PBS, pH 7.2) for three times. Finally, the infected cells were harvested in the proper method to perform the following experiments. To transfect the plasmid, cells were transiently transfected using 1.5 μg plasmid in six-well plates and 0.5 μg in 24-well plates mixed with 6 μL and 2 μL of liposome (Roche, Mannheim, Germany), and incubated for 8 h with serum-free media without antibiotics. Cells were cultured in the presence of plasmids for another 40 h in fresh completed medium before other treatments.

### 2.3. SDS-PAGE and Western Blot

Sodium dodecyl sulfate polyacrylamide gel electrophoresis (SDS-PAGE) and immunoblot analysis (Western blotting) were performed for ATG8 and VP39 at diverse infection times and p62-Flag was treated as previously described above. All of the samples were lysed using Western and immunol precipitation (IP) lysis buffer (P0013, Beyotime, Beijing, China) containing phenylmethanesulfonyl fluoride (PMSF). The concentration of protein samples were detected using BCA Protein Assay Kit (Beyotime, P0012S) according to the manufacturers’ instructions. Then, we adjusted the sample volume according to the concentration for the electrophoretic experiment. The clarified lysates pelleted by centrifugation were boiled in 5× SDS-PAGE loading buffer for 10 min, and they were subjected to 12% SDS-PAGE gel and then electrotransferred onto polyvinylidene fluoride (PVDF) membranes. The membranes were blocked for 1 h, and incubated with the following primary antibodies for 2 h: rabbit anti-ATG8 antibody (1:1000, Youke, Shanghai, China); rabbit anti-VP39 antibody (1:1000, Youke, Shanghai, China); mouse Flag-tag antibody (1:1000, M20008, Abmart, Shanghai, China) and α-tubulin rabbit polyclonal antibody (AF0001, Beyotime, Beijing, China). The tubulin band indicated the total protein loading level. Horseradish peroxidase (HRP)-labeled goat anti-mouse or goat anti-rabbit IgG (H + L) (1:5000, Beyotime, A0216 or A0208, Beijing, China) was used as the secondary antibody for 1 h at room temperature. The protein bands were visualized using enhanced chemiluminescence (ECL) reagents (Roche, Mannheim, Germany). Blue PlusTM protein marker (14–120 KD) and EasySee^TM^ western marker (25–90 KD, TransGene Biotech, Beijing, China) were used as the protein markers.

### 2.4. Transmission Electron Microscopy

Transmission electron microscopy (TEM) was used to observe autophagosomes as described previously [20]. Specifically, BmN-SWU1 cells were collected at six and 48 hours post infection (hpi) with BmNPV. Then, the samples were fixed in 2.5% glutaraldehyde for 24 h at 4 °C. Next, they were postfixed in 0.5% osmium tetroxide for 2 h after thoroughly washing with PBS. Finally, the samples were embedded in Epon resin (Hexion, Houston, TX, USA) according to the manufacturer’s recommendations. We cut off 70-nm sections from the fixed, embedded tissues and stained them in Reynold’s lead citrate. A H7650 transmission electron microscope (Hitachi, Tokyo, Japan) was used to observe autolysosomes and autophagosomes [21,22,23].

### 2.5. Confocal Fluorescence Microscopy

For the detection of autophagosomes, cells grown to 80% confluence in 24-well plates were transiently transfected with pIZ-EGFP-ATG8 (EGFP: enhanced Green Fluorescent Protein) before NPV infection, as previously described. In brief, cells were fixed in 4% formaldehyde/PBS, and then washed in PBS/0.1% Triton X-100 (PBST) twice. The cells were rinsed with PBS for 5 min (repeated three times), counterstained with 4’,6-diamidino-2-phenylindole (DAPI) (Sigma-Aldrich, Saint Louis, MO, USA) for 10 min, and examined by fluorescence microscopy (Olympus, Tokyo, Japan). Three wells per treatment and three sites per well were collected. Experiments were performed at least three times.

To detect autophagic flux, the cells were transfected with pIZ-ATG8-EGFP-Mcherry (Mcherry: a monomer fluorescent protein), infected with BmNPV, or left untreated. After fixing and permeabilization, the cells were stained with DAPI as above. After the cells were washed three times with PBS, they were processed for analysis under a confocal microscope (Olympus, Tokyo, Japan).

### 2.6. Real-Time PCR

Total RNA was extracted from BmN-SWU1 cells infected with or without BmNPV and those transfected with *Atgs*-overexpression or knockout plasmids before being infected with BmNPV. Real-time PCR was performed using iTaq^TM^ Universal SYBR^®^ Green Supermix (Bio-Rad, Berkeley, CA, USA) in accordance with the manufacturers’ instructions. The CFX Connect Real-Time PCR Detection System (Bio-Rad, Berkeley, CA, USA) was use to run the following programs: 95 °C for three minutes, followed by 44 cycles of 95 °C for 10 s, 60 °C for 30 s, and 95 °C for 10 s. The primers are listed in Table 2 and *ribosomal protein L3* (*BmRPL3* NM_001126254) was used as an internal control. Each sample was analyzed at least three times for the expression of the gene of interest.

### 2.7. Knockout Efficiency Analysis

Total DNA was extracted from the cells under various treatments using a Wizard Genomic DNA extraction kit (Promega, Madison, WI, USA). Genomic fragments, including the target site, were amplified by PCR and ligated into the pMD19-T vector for sequencing using M13 primers. All of the primers used to detect the target gene are presented as *Atgs*-T in Table 2.

### 2.8. Statistical Analysis

Statistical analyses were performed using GraphPad Prism 5.0 software (GraphPad Software, La Jolla, CA, USA). Student’s *t*-test was used to analyze the significance. *p* < 0.05 was considered to be significantly different and *p* < 0.01 was considered to be extremely significantly different.

## 3. Results

### 3.1. BmNPV Infection Triggered Autophagy in BmN-SWU1 Cells

**Figure 1:**
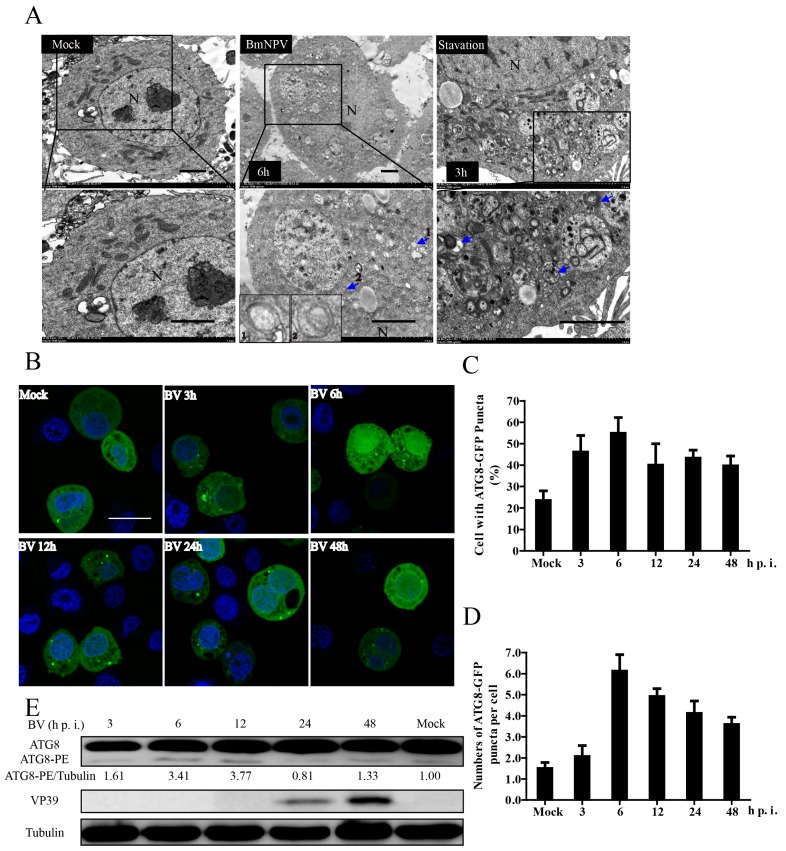
*Bombyx mori* nuclear polyhedrosis virus (BmNPV) infection triggers autophagy in BmN-SWU1 cells. (**A**) BmNPV induced the formation of autophagic bodies by transmission electron microscopy (TEM) analysis. Mock BmN-SWU1 cells as a negative control showed no double-membrane autophagic vesicles. Cells infected with BmNPV at 6 hours post infection (hpi) and starvation treatment had many autophagosome-like structures, which are indicated by the blue arrows, and BV particles at 48 hpi are indicated by the red ones. Scale bars: 2 μm. Starvation-treated BmN-SWU1 cells were set as a positive control; (**B**) BmATG8-Enhanced Green Fluorescent Protein (EGFP) puncta increased post-BmNPV infection through observation by confocal microscopy. The aggregation of autophagosomes is shown as green puncta in the cells that were transfected with ATG8-EGFP followed by BmNPV infection or untreated mock as a negative control. The cell nuclei were stained with 4′,6-diamidino-2-phenylindole (DAPI). Scale bar: 20 μm; (**C**,**D**) percentage of cells with green puncta and the average number of puncta per cell from statistical data of figure (**B**); (**E**) the lapidated form of ATG8 (ATG8-PE or ATG8-II) was detected by Western blotting. The intensity band ratios of ATG8-II/tubulin are shown in the middle space between the bands. The values represent the mean ± SD of three independent experiments.

The process of BmNPV infection and the interaction with its unique host silkworm are very complex. Here, we chose BmNPV to explore if it can trigger autophagy in infected BmN-SWU1 cells. Autophagy is usually detected by three assays: electron microscopy, fluorescent GFP-LC3 puncta, or as lipidated LC3 by immunoblot [24]. First, we monitored autophagy by TEM at 6 hpi, the most convincing approach for monitoring autophagosome induction [25]. As shown in Figure 1A, double-membrane vesicles were rarely observed in mock cells. In contrast, many double-membrane vesicles appeared in BmNPV-infected cells, suggesting that BmNPV infection could trigger the production of autophagosomes in BmN-SWU1 cells (Figure 1A).

GFP-LC3 is an autophagosome-specific membrane marker that is detected in punctate upon autophagosome induction in both mammalian and insect systems [26,27,28]. Thus, to further verify the results of TEM, the formation of EGFP-ATG8 (LC3 homologue of human) dots was investigated in pEGFP-ATG8-transfected cells before they were infected with BmNPV or not. As presented in Figure 1B, a considerable number of EGFP-ATG8 punctate accumulated in BmNPV infected cells compared with the mock. Statistical results also showed the percentage of cells with EGFP-ATG8 punctate increased to over 55% at 6 hpi compared to lower than 25% in the control (Figure 1C). In addition, the number of EGFP-ATG8 punctates per cell increased obviously as shown in Figure 1D. Curiously, autophagosomes seemed to decrease with the duration of infection, combining as noted by the results of TEM and fluorescence. Thus, we speculated that degradation of the autophagosomes or the levels of autophagic flux increased with the time post infection.

The lipidated form of LC3 (LC3-II) is a hallmark of autophagy induction, and it is widely used to determine the presence of autophagy. In our study, the lipidated form of ATG8 (ATG8-PE) was monitored by immunoblotting at the appropriate time after BmNPV infection. As shown in Figure 1E, the transformation from ATG8 to ATG8-PE was significantly greater in the BmNPV-infected cells relative to the mock, which indicated that the formation of autophagic membranes increased. The ratio of ATG8-PE to tubulin (as an indicator) was significantly increased from 1 without infection, to the peak, 3.77, at 12 hpi. Meanwhile, the polyantibodies of BmNPV vp39 protein (a late expressed capsid protein) were used to track infection progression. By combining the above results, we confirmed that BmNPV infection could trigger autophagy in BmN-SWU1 cells.

### 3.2. BmNPV Infection Increased the Levels of Autophagic Flux

**Figure 2:**
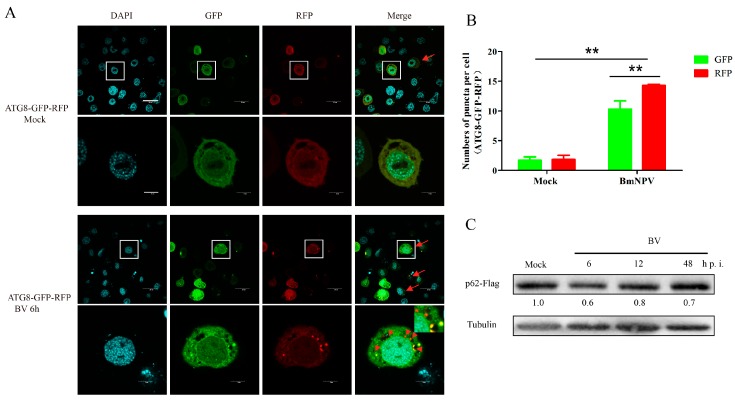
Autophagic flux measurement in BmN-SWU1 cells infected with BmNPV. (**A**) ATG8-GFP-RFP (RFP: Red Fluorescent Protein) autophagy tandem sensor to monitor the autophagic flux by confocal microscope. BmN-SWU1 cells transfected with ATG8-GFP-RFP were infected with or without BmNPV. Cells with red and green clusters not completely coincident are indicated by the red arrows and the red clusters only are marked by red arrowheads. The cells in the white frame are further enlarged below. The cell nuclei are stained with DAPI. Scale bars: 20 μm upper and 5 μm below; (**B**) representative graphs are shown exhibiting the number of GFP and RFP puncta per cell (mean ± SD) from three independent experiments. Statistical significance was analyzed with Student’s *t*-test (** *p* < 0.01); and (**C**) p62/SQSTM1 (p62) degradation was tested by Western blotting. The overexpressed p62-Flag in virus-infected and mock-infected BmN-SWU1 cells was monitored using an anti-Flag antibody. The intensity band ratios of p62/tubulin are shown in the middle space between the bands.

Autophagic flux is a continuous and complete process of autophagy. As we have proven above, BmNPV infection triggered obvious autophagy in the early period of infection, but then it relatively declined. To investigate this phenomenon, we used the ATG8-GFP-RFP autophagy tandem sensor to monitor the autophagic flux as tagRFP pKa ≤ 4 while EGFP pKa = 6.15, which meant that the green fluorescence was quenched while the red fluorescence persisted when the autolysosomes were formed to degrade the autophagosomes. [29,30]. We found that the red and green fluorescence were almost completely coincident in normal cells (Figure 2A). In the BmNPV-infected cells, we observed obvious red clusters without overlapping green fluorescence, which represented the fusion body of lysosomes and autophagosomes (Figure 2A). The statistical results showed that there were significantly more red fluorescence dots than green in the BmNPV-infected cells (Figure 2B).

p62 (SQSTM1) is a selective autophagy receptor and an indicator to assess autophagic flux. P62 can selectively target specific cargoes for autophagy and it will be degraded with its cargoes by the autolysosomes when autophagic flux occurs [31]. Therefore, we detected the degradation level of the p62 protein by transfecting cells with pIZ-*p62*-Flag. Immunoblotting revealed significant progressive degradation of p62 in the BmNPV-infected cells compared with the mock (Figure 2C), suggesting that autophagosomes were able to fuse with lysosomes to degrade the cargos. The ratios of p62 to tubulin shown below indicated that p62 reduction was notable. In conclusion, BmNPV infection increased the levels of autophagic flux.

### 3.3. BmNPV Infection Caused Expression Level Changes of Autophagy-Related Genes

**Figure 3:**
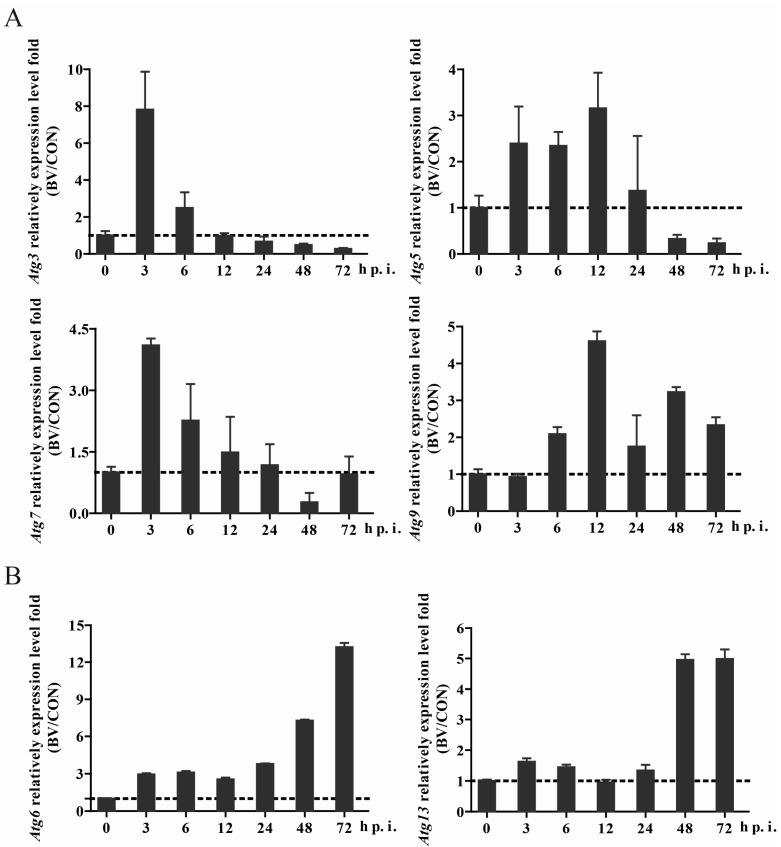
BmNPV infection caused an expression level change of autophagy-related genes. *Atg* gene expression was revealed by qPCR along with the extension of viral infection. The dotted lines represent the expression level of the gene in normal cells. Representative genes showing similar expression trends are put together ((**A**): *Atg3*, *5*, *7*, and *9* and (**B**): *Atg6*, and *13*). Others are shown in Figure S2.

Fifteen putative autophagy-related genes have been identified in the *Bombyx* genome, namely, *Atg1*, *Atg2*, *Atg3*, *Atg4*, *Atg5*, *Atg6* (ortholog of yeast *VPS30* and human *BECN1*), *Atg7*, *Atg8*, *Atg9*, *Atg11*, *Atg12*, *Atg13*, *Atg14*, *Atg16*, and *Atg18* [22,32]. Through the prediction of the domains of the proteins encoded by these *Atg* genes, we found that all of them showed conserved domains and functions of their mammalian homologs (Figure S1 and Table S1). Given autophagy occurred necessarily accompanied by the changes of autophagy-related genes, we would like to know if they were changed and the infective stage of change while BmNPV induced autophagy. Thus, we detected the expression level of almost all of the autophagy-related genes. Surprisingly, we discovered that the expression level of most *Atg* genes (*Atg1*, *2*, *3*, *4*, *5*, *7*, *8*, *11*, *12*, and *16*) exhibited a tendency to increase before 24 hpi and then decrease (Figure 3A and Figure S2), while *Atg6* and *Atg13* were also up-regulated in the early stage of viral infection but more pronounced after 24 h post infection (Figure 3B). Additionally, *Atg9* and *Atg18* also maintained an upward trend, but a few fluctuations occurred (Figure 3 and Figure S2). All of the above results suggested that BmNPV induced autophagy-related gene changes due to autophagy, or perhaps other related functions.

### 3.4. Atg Genes Influence the Process of BmNPV Infection

**Figure 4:**
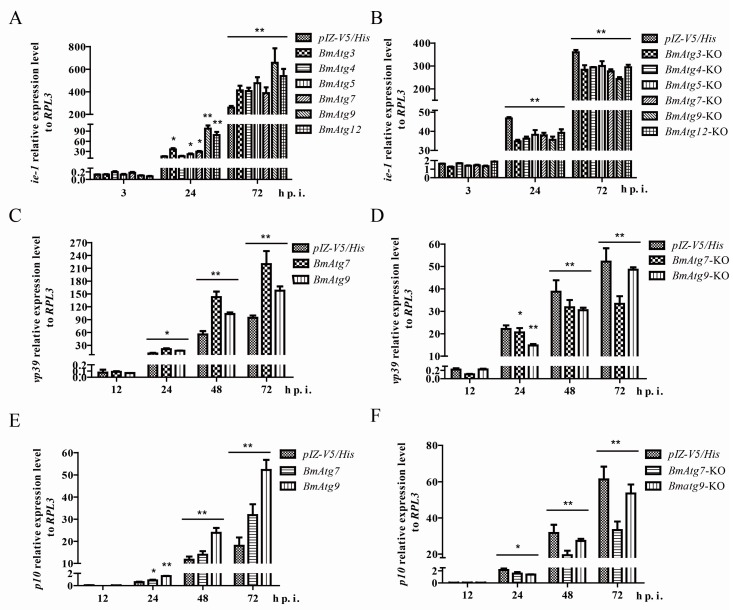
Autophagy-related genes influence BmNPV genes expression. (**A**) *Atgs* overexpression promoted BmNPV *ie-1* relative mRNA level compared with the control; (**B**) knockdown of autophagy genes by CRISPR/Cas9 gene editing system leads to a decrease in the BmNPV *ie-1* mRNA level as compared to control plasmid; (**C**) and (**E**) autophagy-related gene overexpression promoted BmNPV *vp39* and *p10* expression level; and (**D**) and (**F**) knockdown of *Atgs* reduced the expression of *vp39* and *p10*. The values represent the mean ± SD of three independent experiments. Statistical significance was analyzed with Student’s *t*-test (* *p* < 0.05 and ** *p* < 0.01).

To further research what roles autophagy or *Atg* genes played in the process of BmNPV infection, we choose *Atg* genes with the greatest fold change (*Atg3*, *4*, *5*, *7*, *9*, and *12*) to find their relationships with virus infection. We constructed their overexpression and knockdown plasmids (Figure S3). First, we overexpressed those genes before infection, and surprisingly found that the relative expression level of the *ie-1* gene (a BmNPV immediate early gene) increased significantly (Figure 4A). On the contrary, when these genes were knocked down through the CRISPR/Cas9 gene editing system (detection of knock down efficiency is shown in Figure S3), a decreased level of *ie-1* happened significantly (Figure 4B). Then, we divided these genes into three groups according to their position and function in the autophagy pathway: ATG9 (recruitment of PI3K complex); ATG5, ATG7, and ATG12 (ATG16L1 complex); and ATG3, ATG4, and ATG7 (ATG8-II ubiquitin-like conjugation system) [33,34,35,36]. We further selected Atg7 and Atg9 to detect the other two key genes, nucleocapsid protein genes *vp39* and *p10*, and found their expression showed a similar trend (Figure 4C–F). All of the above indicated autophagy and *Atg* genes were essential for the process of virus infection. In conclusion, changes in the expression of autophagy-related genes did impact the effective infection of the virus. The virus may utilize the host’s autophagy mechanism to promote its own infection process.

### 3.5. Atg7 and Atg9 Promote Autophagy Induced by BmNPV In Vitro

**Figure 5:**
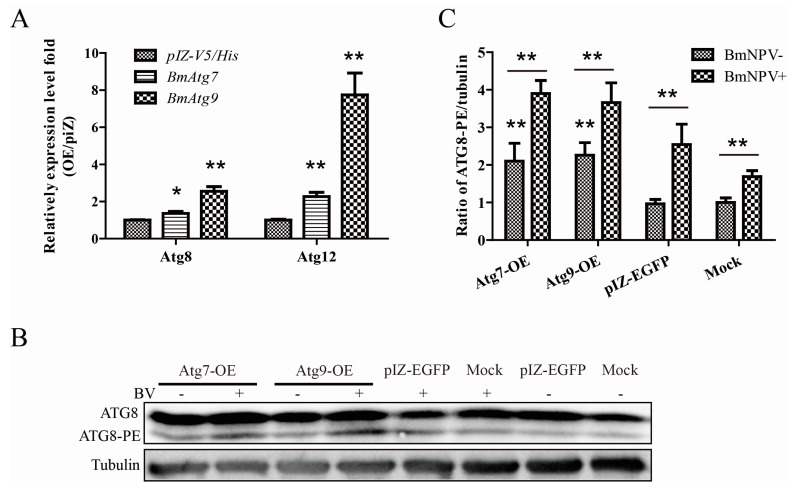
Autophagy-related genes promote autophagy induced by BmNPV in vitro. OE represents overexpressed. (**A**) Overexpressed *Atg7* or *Atg9* up-regulates *Atg8* and *Atg12*; (**B**) the transformation from ATG8 to ATG8-PE was monitored in *Atg7* and *Atg9* overexpressed cells infected with or without BmNPV by immunoblotting; and (**C**) representative results are shown with graphs representing the intensity of ATG8-II/tubulin. The values represent the mean ± SD of three independent experiments. Statistical significance was analyzed with Student’s *t*-test (* *p* < 0.05 and ** *p* < 0.01).

Next, we chose the most remarkable gene, *Atg9*, and the common E1-like enzyme of two ubiquitin-like conjugation systems, *Atg7*, as the objects of the following experiments. To clarify whether autophagy related genes affect the virus by regulating autophagy levels, we detected the relative expression level of downstream genes and the lipidated form of ATG8 (ATG8-PE). As a result, the overexpression of *Atg7* and *Atg9* lead to significant up-regulation of the two ubiquitin-like conjugation system key genes, *Atg8* and *Atg12*, in the formation of autophagy (Figure 5A). Through immunoblotting, the transformation from ATG8 to ATG8-PE was monitored in *Atg7* and *Atg9* overexpressed cells infected with or without BmNPV (Figure 5B). Similarly, we found the ratio of ATG8-PE to tubulin significantly improved in overexpressing *Atgs* than pIZ-EGFP or mock cells with or without BmNPV (Figure 5C).

## 4. Discussion

Viral infection of the host is accompanied by a variety of physiological mechanisms and biological pathways. As one of the most important pathways, autophagy has been widely studied by researchers to understand the interaction mechanism between virus and host. As recently reported, hematopoietic necrosis virus (IHNV) and hepatitis C virus (HCV) infection can induce typical autophagy through different mechanisms. This enables the virus to maximize its replication and attenuate the innate immune responses that it activates [37,38,39]. While the Epstein-Barr virus (EBV) hijacks the autophagic vesicles for its intracellular transportation and enhances viral production [40]. All of these studies imply a complex relationship between viral infection and autophagy in host cells. With a new model system, silkworm and BmNPV, our results are similar to many other viruses in triggering autophagy, such as IHNV and HCV, which we have mentioned before [37,38,39]. However, there exist differences in the stage of inducing autophagy. We found that BmNPV infection induces autophagy at the early stage of infection while it seems to inhibit autophagy at the latter stage, which has not been studied so far. Through a serious of autophagy detection and the mRNA expression level of *Atg* genes (Figure 1, Figure 2 and Figure 3 and Figure S2), we speculated that the early viral infection induces complete autophagy accompanied by an up-regulation of *Atg* gene expression, whereas in the late stage of infection, most *Atg* genes are suppressed, possibly due to the inhibition of autophagy. Meanwhile, autophagy by TEM at 6 hpi supports the above hypothesis. Proteomic analysis showed the up-regulation of the BmATG3 protein in response to BmNPV infection, and *S. exigua* SeATG5 can also be enhanced by the infection of baculovirus at 24 h [41,42]. All of these reports coincide with our results, which further confirm that the virus infection causes autophagy and the up-regulation of *Atg* genes.

Autophagy certainly acts as both an anti-viral and pro-viral pathway, and the roles of autophagy depend on the virus, the cell type, and the cellular environment [43]. Then, what role does autophagy play in the early stage of BmNPV infection? We chose the most obvious changed *Atg* genes post infection to detect their effects on virus infection using an overexpression and knockout technique. As a result, autophagy-related genes significantly promote the viral gene expression just as influenza virus (IV) and rotavirus do [16,44]. However, as for *Autographa californica* nucleopolyhedrovirus (AcMNPV), one of the best known insect baculaviruses, reports have shown that autophagy increased in a *Spodoptera litura* cell line after baculovirus infection of starved cells, but researchers did not observe an increase in *ie-1* transcript levels, as was the case with BmNPV infection in our research [45].

To investigate the possible regulatory mechanisms, we further examined the effects of *Atg7* and *Atg9* on autophagy. Interestingly, when *Atg7* and *Atg9* were overexpressed, there was a higher autophagy level in vitro compared with the control; otherwise *Atg7* and *Atg9* could promote the expression of viral genes as we demonstrated in our studies. Therefore, it is reasonable to assume that the virus may induce autophagy, which is ultimately beneficial to its replication and proliferation, by increasing the expression of some autophagy-related genes. As for how autophagy promotes BmNPV proliferation, we have the following hypotheses. First, autophagy induced by a virus specifically degrades immune-related proteins and attenuates the innate immune responses that it activates to weaken the host defense capabilities, as reported in HCV [39]; second, autophagy triggered by a virus promotes the degradation of a large number of useless proteins or organelles of the host, providing enough energy for virus proliferation; and, finally, a virus initiates autophagy and hijacks this membrane trafficking pathway to transport viral proteins to sites of viral assembly, such as rotavirus and Zika virus (ZIKV) [44,46].

In conclusion, our findings suggest that autophagy triggered by BmNPV is critical to its replication, which indicates that the autophagy pathway in this process may be utilized to sustain viral replication. Furthermore, autophagy-related genes immediately influence the viral infection efficiency in vivo and vitro. Our findings presented here provide novel insights into virus-host interactions and open a new window to the mechanism research of autophagy and viruses. Next, we will further deepen our understanding of the mechanisms driving the interaction between virus infection and host autophagy.

## Figures and Tables

**Table 1:** The plasmids, primers, and singleguide RNAs (sgRNAs) used in this study.

Plasmid Name	Primer Sequence
pIZ/V5-Flag-*BmAtg8*-Enhanced Green Fluorescent Protein (EGFP); pIZ/V5-Flag-*BmAtg8*-EGFP-RFP (RFP: Red Fluorescent Protein)	5′-CGCGGATCCGATTACAAGGATGACGACGATAAGAAATTCCAATACAAAG-3′; 5′-CCGCTCGAGTTAATTTCCATAGACAT-3′
pIZ/V5–Flag-*BmAtg3*	5′-CGGGGTACCATGGATTACAAGGATGACGACGATAAGCAAAGTGTAATAAACACAGTAAAG-3′; 5′-CTAGTCTAGACGGTTAGTTAATCGAGAAATTCTG-3′
pIZ/V5–Flag-*BmAtg4*	5′-CGGGGTACCATGGATTACAAGGATGACGACGATAAGAATGGTTCAAGCGGTT-3′; 5′-CTAGTCTAGAGCGGCTAAACTAAAACAAATTCTTC-3′
pIZ/V5–HA-*BmAtg5*	5′-CGCGGATCCATGTACCCATACGATGTTCCAGATTACGAATGGTTCAAGCGGTT-3′; 5′-CCGCTCGAGCGGTCAGCATAAACACAAATG-3′
pIZ/V5–Flag-*BmAtg7*	5′-CCGGAATTCATGGATTACAAGGATGACGACGATAAGTTGGAAGCACAAAAC-3′; 5′-CTAGTCTAGACGCTAATCTTCTTCATCGGTTAATG-3′
pIZ/V5–Flag-*BmAtg9*	5′-CCGGAATTCATGGATTACAAGGATGACGACGATAAGGCTTACTTCGGAAG-3′; 5′-CTAGTCTAGACTTAGGGACGGTGTAGGAGAG-3′
pIZ/V5–Flag-*BmAtg1*2	5′-GATCCATGGATTACAAGGATGACGACGATAAGAGTGATGAGAAACTTATAGATG-3′; 5′-CCGCTCGAGTCAGCCCCAAGCTTGGCTT-3′
pSL1180-*Cas9*-Flag-sgAtg3; *Atg3*-T	5′-AAGTGCAACACCGGTGTCAAGTAAC-3′; 5′-AAACGTTACTTGACACCGGTGTTGC-3′; 5′-ATGCAAAGTGTAATAAACACAGTAAAG-3′; 5′-TACATCGTCTGTAGCATGGC-3′
pSL1180-*Cas9*-Flag-sgAtg4; *Atg4*-T	5′-AAGTGGAACTAAACGATTTAAATAA-3′; 5′-AAACTTATTTAAATCGTTTAGTTCC-3′; 5′-ATGAATGGTTCAAGCGGTT-3′; 5′-CAACCAAACCGGAGATTCTTTAG-3′
pSL1180-*Cas9*-Flag-sgAtg5; *Atg5*-T	5′-AAGTGGTACTTCGAGAAATATGGGA-3′; 5′-AAACTCCCATATTTCTCGAAGTACC-3′; 5′-ATGGCCAACGACAGGGAG-3′; 5′-CGATGACAAGAGGAAAGTAGC-3′
pSL1180-*Cas9*-Flag-sgAtg7; *Atg7*-T	5′-AAGTGGTGAGGGTATGCCAGAATGA-3′; 5′-AAACTCATTCTGGCATACCCTCACC-3′; 5′-ATGTTGGAAGCACAAAAC-3′; 5′-GTTTTTGTTCATTATTGTACCCATC-3′
pSL1180-*Cas9*-Flag-sgAtg9; *Atg9*-T	5′-AAGTGTCAGCCAATCCACCTAAAGG-3′; 5′-AAACCCTTTAGGTGGATTGGCTGAC-3′; 5′-ATGGCTTACTTCGGAAG-3′; 5′-ACAGTGCACGAGAAATGTTGAG-3′
pSL1180-*Cas9*-Flag-sgAtg12; *Atg12*-T	5′-AAGTGGTTGATGCTGAAAAGCCTAT-3′; 5′-AAACATAGGCTTTTCAGCATCAACC-3′; 5′-ATGAGTGATGAGAAACTTATAGATG-3′; 5′-TCAGCCCCAAGCTTGGCTT-3′

**Table 2:** The primers for RT-PCR used in this study.

Primers Name	Primer Sequence(RT-PCR)
*BmAtg1*	5′-CATCGTCCACCGTGACTTGA-3′; 5′-GTCTGCTTTGGCGTCGTATTT-3′
*BmAtg2*	5′-GACGACTCACCGATTTACTTCAGA-3′; 5′-CTCAGTGCCCAACAATCCAAG-3′
*BmAtg3*	5′-AACTCAAAGCCGATAAGAAACA-3′; 5′-TTTTAGCGTGATCTTGGGAC-3′
*BmAtg4*	5′-TACCTCAGGGTGTATCATCA-3′; 5′-TAAGTCTGTATCGCTGTCTTG-3′
*BmAtg5*	5′-AAGTTCCCTGAAGACATTCT-3′; 5′-ATTTTGTAATCCAAGCCATA-3′
*BmAtg6*	5′-GGGCTTTTGTCTTCCGTA-3′; 5′-TGTGGCTCAGATTTGTCCT-3′
*BmAtg7*	5′-GAGGCGAGATGGCTGC-3′; 5′-CGAGGTGCTAATTCCGTG-3′
*BmAtg8*	5′-AAGGCTAGGCTTGGAGAC-3′; 5′-CAGATGTGGGTGGAATGA-3′
*BmAtg9*	5′-TTTAGGTGGATTGGCTGA-3′; 5′-TTGGCACTTCGTGGATG-3′
*BmAtg11*	5′-TAAGTCCCATAGTAGAGC-3′; 5′-ACAAACTTCCACTTCAT-3′
*BmAtg12*	5′-AGACGCAGAGCCAATCA-3′; 5′-AACTCCATAATCCATCCAATA-3′
*BmAtg13*	5′-AGAGTTTACAGTGGCGAG-3′; 5′-TGTCTGAACGGTTAGGAG-3′
*BmAtg16*	5′-CACATTCGGTAGAGTTAGTTTCGG-3′; 5′-CGGCATACACTTCTCCATCGT-3′
*BmAtg18*	5′-TTGCTGTTGGCGGTCT-3′; 5′-CAAGCGATATGGCGGA-3′
*BmNPVie-1*	5′-AAGAAGGAGGACGGCAGCAT-3′; 5′-ATCTCGCCAGAAATCCAATAAAAC-3′
*BmNPVvp39*	5′-CTAATGCCCGTGGGTATGG-3′; 5′-TTGATGAGGTGGCTGTTGC-3′
*BmNPVp10*	5′-TAGACGCCATTGCGGAAA-3′; 5′-CGGGCAAACCGTCCAAA-3′
*BmRPL3*	5′-CGGTGTTGTTGGATACATTGAG-3′; 5′-GCTCATCCTGCCATTTCTTACT-3′

## Supplementary Materials

Supplementary materials can be found at www.mdpi.com/1999-4915/10/1/14/s1.
