# Supplementary material for: Bombyx mori Nuclear Polyhedrosis Virus (BmNPV) Induces Host Cell Autophagy to Benefit Infection
Source: Viruses. 2017 Dec 30;10(1):14. doi: 10.3390/v10010014 (PMC5795427; doi:10.3390/v10010014)
Supplement: Supplementary file 1 [file viruses-10-00014-s001.pdf]

Supplementary Materials

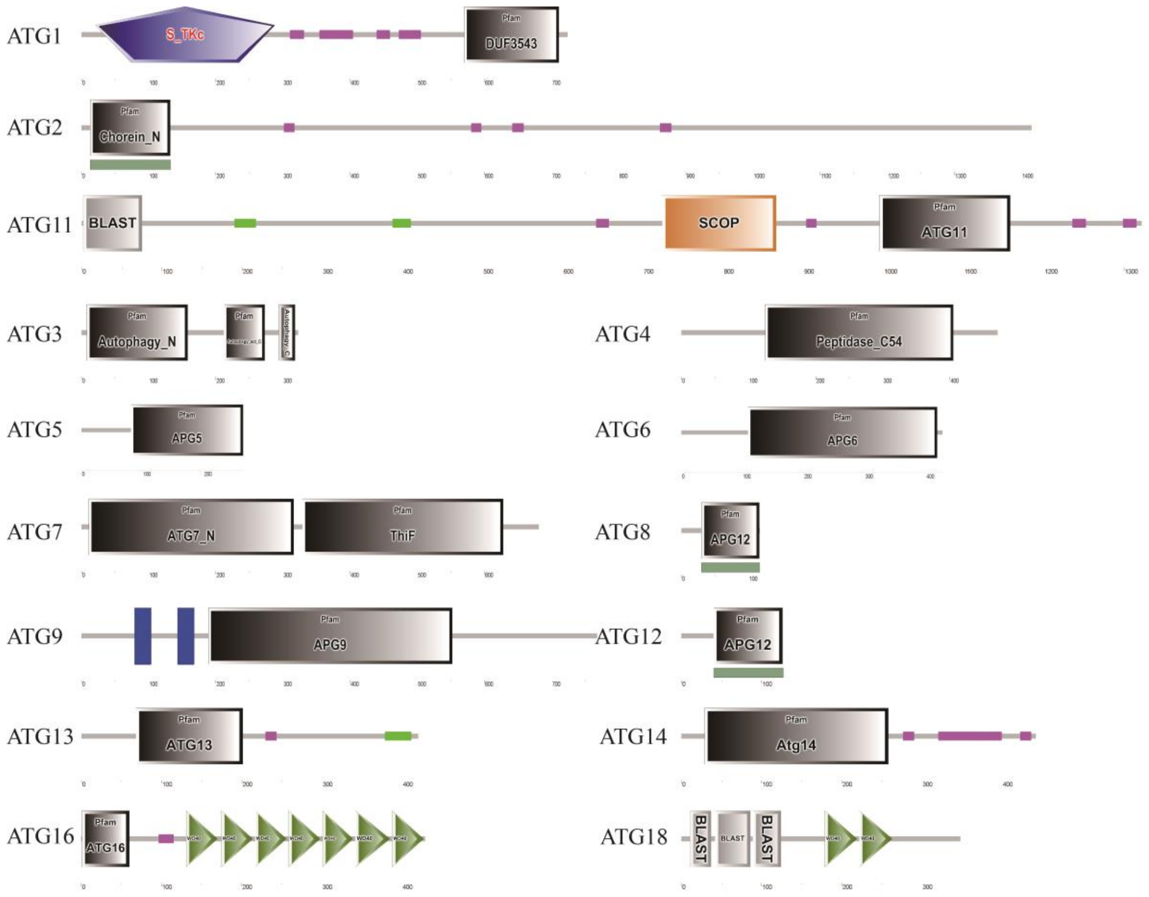

Figure S1. Fifteen autophagy-related genes in *Bombyx mori* have their conserved domains as predicted by SMART (http://smart.embl-heidelberg.de/).

Table S1. Function of autophagy-related genes.

Atg genes	Aliases	Complexes	Function
Atg1	ULK	ULK complex	Phosphorylates Atg9(1) and Atg6(2) as a Ser/Thr kinase; regulates autophagy
Atg11	Atg17/FIP200	(induction of	Mediates mTor signaling to autophagy with ULK-Atg13(3)
Atg13	APG13	autophagy)	Interaction with Atg1(4) and regulates localization to the PAS(5)
Atg9	APG9/CVT6	ATG9L complex	Recruitment of PI3K complex(6) and Atg proteins(1)
Atg6	VPS30/Beclin1	ATG14L complex(apg-specific	Component of class III PI3K complexes(7); binds Atg14 and Rubicon(8, 9)
Atg14	APG14	PI(3)K complex)	Localizes on the ER to induce autophagy(10)
Atg2	APG2	Atg2-Atg18 complex	Localizes to some membranous structure and is essential for autophagy(11)
Atg18	WIPI		Binds to Atg16L1 and recruits ATG16 complex to APS(12)
Atg5	APG5	Atg16L1 complex (as	Conjugates to Atg12 to form ATG16L1 complex(13); involves in sequestration(14)
Atg7	APG7	E3-like enzyme for	Mediates the conjugation of Atg5 and Atg12 as an E1-like enzyme(15)
Atg12	APG12	LC3-PE)	Conjugates to Atg5 to form ATG16L1 complex(13)
Atg16	APG16/CVT11		Forms a 2:2:2 complex with Atg12-Atg5 as E3-like enzyme for LC3-PE
Atg8	LC3	ATG8-II ubiquitin-like	Marker and closure of isolated membrane(16); quantification of autophagy activity(17)
Atg3		conjugation system	Mediates LC3 conjugating to PE as a E2-like enzyme with Atg7(18)
Atg4			Cleaves the C-terminal arginine of Atg8(19) and Mediates LC3 deconjugating from the outer autophagosomal and LAP phagosome membrane(20)
Atg7			Mediates LC3 conjugating to PE as a E1-like enzyme with Atg3(18)

1. Papinski D, Schuschnig M, Reiter W, et al. Early steps in autophagy depend on direct phosphorylation of Atg9 by the Atg1 kinase. Molecular cell. 2014;53(3):471-83. doi:10.1016/j.molcel.2013.12.011
2. Russell RC, Tian Y, Yuan H, et al. ULK1 induces autophagy by phosphorylating Beclin-1 and activating VPS34 lipid kinase. Nature cell biology. 2013;15(7):741-50. doi:10.1038/ncb2757
3. Jung CH, Jun CB, Ro SH, et al. ULK-Atg13-FIP200 complexes mediate mTOR signaling to the autophagy machinery. Molecular biology of the cell. 2009;20(7):1992-2003. doi:10.1091/mbc.E08-12-1249
4. Kamada Y, Yoshino K, Kondo C, et al. Tor directly controls the Atg1 kinase complex to regulate autophagy. Molecular and cellular biology. 2010;30(4):1049-58. doi:10.1128/MCB.01344-09
5. Stephan JS, Yeh YY, Ramachandran V, Deminoff SJ, Herman PK. The Tor and PKA signaling pathways independently target the Atg1/Atg13 protein kinase complex to control autophagy. Proceedings of the National Academy of Sciences of the United States of America. 2009;106(40):17049-54. doi:10.1073/pnas.0903316106
6. Kageyama S, Omori H, Saitoh T, et al. The LC3 recruitment mechanism is separate from Atg9L1-dependent membrane formation in the autophagic response against Salmonella. Molecular biology of the cell. 2011;22(13):2290-300. doi:10.1091/mbc.E10-11-0893
7. Kihara A, Noda T, Ishihara N, Ohsumi Y. Two distinct Vps34 phosphatidylinositol 3-kinase complexes function in autophagy and carboxypeptidase Y sorting in Saccharomyces cerevisiae. The Journal of cell biology. 2001;152(3):519-30.
8. Matsunaga K, Saitoh T, Tabata K, et al. Two Beclin 1-binding proteins, Atg14L and Rubicon, reciprocally regulate autophagy at different stages. Nature cell biology. 2009;11(4):385-96. doi:10.1038/ncb1846
9. Zhong Y, Wang QJ, Li X, et al. Distinct regulation of autophagic activity by Atg14L and Rubicon associated with Beclin 1-phosphatidylinositol-3-kinase complex. Nature cell biology. 2009;11(4):468-76.

doi:10.1038/ncb1854
10. Matsunaga K, Morita E, Saitoh T, et al. Autophagy requires endoplasmic reticulum targeting of the PI3-kinase complex via Atg14L. The Journal of cell biology. 2010;190(4):511-21. doi:10.1083/jcb.200911141
11. Shintani T, Suzuki K, Kamada Y, Noda T, Ohsumi Y. Apg2p functions in autophagosome formation on the perivacuolar structure. The Journal of biological chemistry. 2001;276(32):30452-60. doi:10.1074/jbc.M102346200
12. Dooley HC, Razi M, Polson HE, Girardin SE, Wilson MI, Tooze SA. WIPI2 links LC3 conjugation with PI3P, autophagosome formation, and pathogen clearance by recruiting Atg12-5-16L1. Molecular cell. 2014;55(2):238-52. doi:10.1016/j.molcel.2014.05.021
13. Matsushita M, Suzuki NN, Obara K, Fujioka Y, Ohsumi Y, Inagaki F. Structure of Atg5.Atg16, a complex essential for autophagy. The Journal of biological chemistry. 2007;282(9):6763-72. doi:10.1074/jbc.M609876200
14. George MD, Baba M, Scott SV, et al. Apg5p functions in the sequestration step in the cytoplasm-to-vacuole targeting and macroautophagy pathways. Molecular biology of the cell. 2000;11(3):969-82.
15. Kim J, Dalton VM, Eggerton KP, Scott SV, Klionsky DJ. Apg7p/Cvt2p is required for the cytoplasm-to-vacuole targeting, macroautophagy, and peroxisome degradation pathways. Molecular biology of the cell. 1999;10(5):1337-51.
16. Fujita N, Hayashi-Nishino M, Fukumoto H, et al. An Atg4B mutant hampers the lipidation of LC3 paralogues and causes defects in autophagosome closure. Molecular biology of the cell. 2008;19(11):4651-9. doi:10.1091/mbc.E08-03-0312
17. Kabeya Y, Mizushima N, Ueno T, et al. LC3, a mammalian homologue of yeast Apg8p, is localized in autophagosome membranes after processing. The EMBO journal. 2000;19(21):5720-8. doi:10.1093/emboj/19.21.5720
18. Nakatogawa H, Suzuki K, Kamada Y, Ohsumi Y. Dynamics and diversity in autophagy mechanisms: lessons from yeast. Nature reviews. Molecular cell biology. 2009;10(7):458-67. doi:10.1038/nrm2708
19. Kirisako T, Ichimura Y, Okada H, et al. The reversible modification regulates the membrane-binding state of Apg8/Aut7 essential for autophagy and the cytoplasm to vacuole targeting pathway. The Journal of cell biology. 2000;151(2):263-76.
20. Nakatogawa H, Ishii J, Asai E, Ohsumi Y. Atg4 recycles inappropriately lipidated Atg8 to promote autophagosome biogenesis. Autophagy. 2012;8(2):177-86. doi:10.4161/auto.8.2.18373

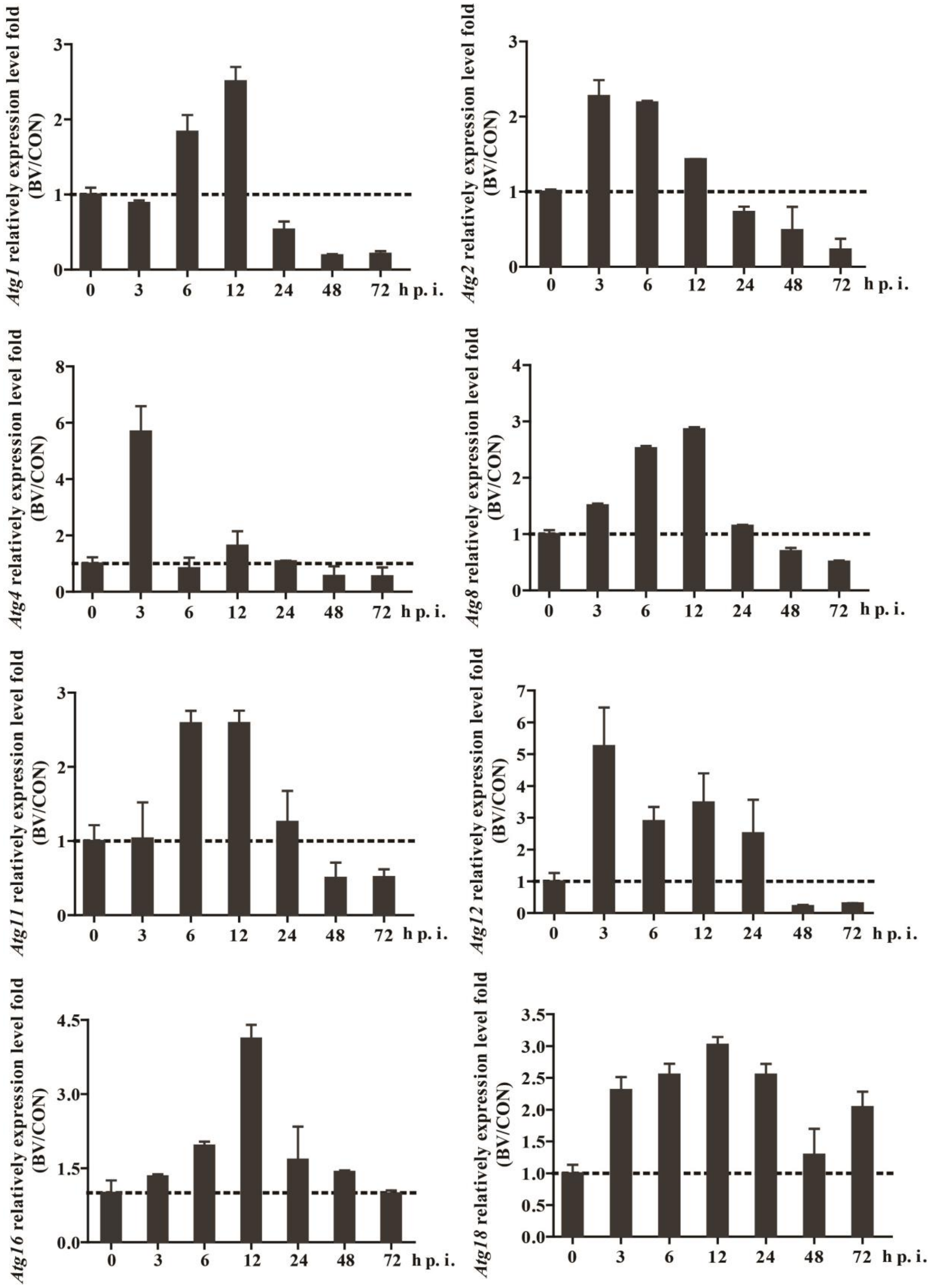

Figure S2. BmNPV infection caused the expression level change of autophagy-related genes. *Atg1*, *2*, *4*, *8*, *11*, *12*, *16*, and *18* expression-level fold changes to control are listed after infection.

ATG3 22.2%

```
TGGCCGGTTACTTGACACCGGTGTTGAAGGTACGTTTATCACGATCATTATTAATTACATACATATTGAAATT
TGGCCGGTT--------------------GTGTTGAAGGTACGTTTATCACGATCATTATTAATTACATACATATTGAAATT
TGGCCGG----------------------------------------------------------------------------------------------------CATACATATTGAAATT
TGGCCG---------------------------------------------------------------------------------------------------------------------------------------
TGGCCGGTT-----TTGACACCGGTGTTGAAGGTACGTTTATCACGATCATTATTAATTACATACATATTGAAATT
TGGCCGG----------------------------------------------------------------------------------------------------------------------TTGAAATT
TGGCCGGTT------------------------------------------------------------------------------------------------------------------TTGAAATT
```

ATG4 33%

```
CTCATACCCCACTGACAAACGCTCGAGACAATGCTGAAGATTCAATTGA
CTCATACCCCAC--GACAAACGCTCGAGACAATGCTGAAGATTCAATTGA
CTCATACCCCAC--------AAACGCTCGAGACAATGCTGAAGATTCAATTGA
CTCATACCC--------------------CGCTCGAGACAATGCTGAAGATTCAATTGA
CTCATACCCCACTG-------------------CGAGACAATGCTGAAGATTCAATTGA
CTCATACCCCAC----------AACGCTCGAGACAATGCTGAAGATTCAATTGA
CTCATACCCCAC-------------------------------ACAATGCTGAAGATTCAATTGA
```

ATG5 25%

```
TTTTCCATCCCATATTTCTCGAAGTACCTCCCTGTCGTTGGCCATAAGGG
TTTTCCATCCCAT----TTCTCGAAGTACCTCCCTGTCGTTGGCC----AAGGG
TTTTCCATCC--A--ATTTCTCGAAGTACCTCCCTGTCGTTGGCCATAAGGG
TTTTCCATCC--A----- ----------GAAGTACCTCCCTGTCGTTGGCCATAAGGG
TTTTCCAT--------------------------------------CTCCCTGTCGTTGGCCATAAGGG
TTTTCCATC---------------------GAAGTACCTCCCTGTCGTTGGCCATAAGGG
```

ATG7 22.2%

```
AATATTTGTTTGGTAGTTTCCTTTAATTTGTCTACTTCTAATTTCATTTCAGTGAGGGTATGCCAGAATGA
AATATTTGTTTGGTAGTTTCCTTTAATTTGTCTACTTCTAATTTCATT---------- ----------------------------- -GA
AATATTTGTTTGGTAGTTTCCTTTAATTTGTCTTG---------------------- ----------------------------------------------  A
AATATTTGTTTGGTAGTTTCCT------------------------------------------------------------------------------------------ATGA
AATATTTGTTTGGTAGTTTCCTTTAATTTGTCTACTTCTAATTTCATT--------------------------------------------GA
AATATTTGTTTGGTAGTTTCCTTTAATTTGTCTACTTCTATG----------------------------------------------------------A
AATATTTGTTTGGTAGTTTCCTTTAATTTGTCTACTTCTAATTTCATT--------------------------------------------TGA
```

ATG9 25%

```
CCTTTAGGTGGATTGGCTGACAATGTGAACATAACGTCGCCTAAAGGCAGTGTAGATGCTGATCATGAAGATTACAGTAAGGTGATCATCCACGAAGTGC
CCTT--AGGTGGATTGGCTGACAATGTGAACATAACGTCGCCTAAAGGCAGTGTAGATGCTGATCATGAAGATTACAGTAAGGTGATCATCCACGAAGTGC
CCTT----------GGATTGGCTGACAATGTGAACATAACGTCGCCTAAAGGCAGTGTAGATGCTGATCATGAAGATTACAGTAAGGTGATCATCCACGAAGTGC
CTTTAG-----TGGATTGGCTGACAATGTGAACATAACGTCGC-TAAAGGCAGTGTAGATGCTGATCATGAAGATTACAGTAAGGTGATCATCCACGAAGTGC
CCTGGAT------------TGGCTGACAATGTGAACATAACGTCGCCTAAAGGCAGTGTAGATGCTGATCATGAAGATTACAGTAAGGTGATCATCCACGAAGTGC
CCTAAG----------------------------------------------------------------------------------------------------------------------------------------------------------------------------------------------TGC
```

ATG12 42%

```
AAACTCCATAATCCATCCAATAGGCTTTTCAGCATCAACAGCCCATTTTTTTTTCTTC
AAACTCCATAATCCATCCAATA-----CTTTTCAGCATCAACAGCCCATTTTTTTT--CTTC
AAACTCCATAATCCATCCAATA--------------------CATCAACAGCCCATTTTTTTTTCTTC
AAACTCCATAATCCATCCAATA---GCTTTTCAGCATCAACAGCCCATTTTTTTT-CTTC
AAACTCCATAATCCATCCAATA--------------------------CAACAGCCCATTTTTTTTTCTTC
AAACTCCATAATCCATCCAATAAG----------------CATCAACAGCCCATTTTTTTTTCTTC
AAACTCCATAATCCATCCGATA----------------------ATCAACAGCCCATTTTTTTTTCTTC
AAACTCCATAATCCATCCAAT-----------------------------------------------ATTTTTTTTTCTTC
AAACT-----------------------------------GGCTTTTCAGCATCAACAGCCCATTTTTTTTTCTTC
```

Figure S3. Knockout efficiency of autophagy-related genes was analyzed through PCR and ligated into the pMD19-T vector for sequencing using M13 primers. *Atg3*, *4*, *5*, *7*, *9*, and *12* are knocked down through the CRISPR/Cas9 gene editing system.
